# Fearsome Creatures and Nature's Gothic

**DOI:** 10.3201/eid1104.AC1104

**Published:** 2005-04

**Authors:** Polyxeni Potter

**Affiliations:** * Centers for Disease Control and Prevention, Atlanta, Georgia, USA

**Keywords:** Dürer, Renaissance, Gothic, stag beetle, ticks, meningoencephalitis, bed bugs

**Figure:**
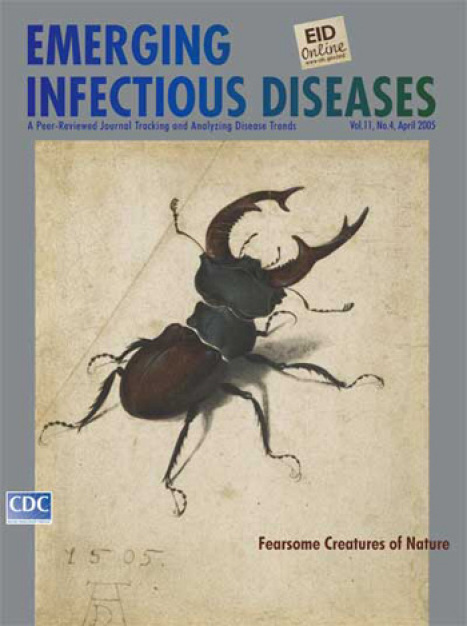
**Albrecht Dürer (1471–1528). Stag Beetle (1505)** Watercolor and gouache; upper left corner added with tip of left antenna painted in by a later hand (14.1 cm x 11.4 cm). The J. Paul Getty Museum, Los Angeles, California, USA

"In Venice, I am treated as a nobleman…. I really am somebody, whereas at home I am just a hack," (*1*) said Albrecht Dürer of his life and studies abroad. During his travels, he came under the influence of Italian Renaissance, which had a transforming effect on the way he viewed art. Challenging his Gothic roots as well as his use of color, travel opened the door to the work of Leonardo da Vinci, engraver Andrea Mantegna, and founder of the Venetian school of painting, Giovanni Bellini, "…the best painter of them all" (*2*).

Dürer was born in Nürnberg, Germany, a bustling humanist center of the Reformation. The third of 18 children in a Hungarian family of goldsmiths, he apprenticed as metalworker from a very young age, his exacting skills evident later in the unparalleled detail and precision of his famed woodcuts, engravings, and etchings. Intellectually gifted and versatile, he was as comfortable with mathematics and writing as with art. His confidence and charisma were immortalized in a series of striking self-portraits and captured in his epitaph: "Whatever was mortal in Albrecht Dürer lies beneath this mound" (*3*).

Synthesizing Gothic traditions of the North with theories and practices of Italy, Dürer flourished as exponent of Northern Renaissance (*4*). He became an exceptional painter, but his greatest impact was on printmaking, which he elevated to art form. Introducing new tonal and dramatic features to graphic images, he increased their conceptual scope and intensity as well as technical perfection.

Even before his travels to Italy, which during this period enjoyed a revival of mathematics, Dürer came to believe that "…art must be based upon science—in particular, upon mathematics, as the most exact, logical, and graphically constructive of the sciences" (*5*). He studied geometric principles, from Pythagoras, Plato, and Euclid to Piero della Francesca, Luca Pacioli, and da Vinci. Specifically, Dürer was interested in Platonic and Archimedean solids and the golden mean and how these mathematical concepts influenced proportion and geometric ratios in art, affecting beauty and meaning.

Dürer had access to Europe's best-known theologians and scholars, including Erasmus, and his diverse portfolio contained portraits of Holy Roman Emperors Maximilian I and Charles V. Many of his works had religious themes, but he was also partial to nature. In his Treatise on Proportion, he commented, "Life in nature makes us recognize the truth of these things, so look at it diligently, follow it, and do not turn away from nature to your own thoughts…. For, verily, art is embedded in nature; whoever can draw her out, has her" (*6*).

Within larger themes or alone in spectacular nature scenes, exotic animals were a large part of Dürer's work. In Rhinoceros, one of his most popular animal engravings, the artist meticulously detailed a creature he had never seen in an image that served as scientific model for the species for more than two centuries (*7*). In other works, insects and beasts symbolized doubt, temptation, or other failings and tormented people, as if in contest for the human soul. In Christ in Limbo, the tormentor was a half-human pig (*8*).

Among the best-known of Dürer's nature works, The Stag Beetle on this month's cover is startling, and not only for its artistic presentation. Insects, though much in line with the artist's Renaissance interest in nature, were thought the lowest of creatures by his contemporaries, hardly suitable focal points for period art.

The Stag Beetle is not the scientific study of a curious bug. It is a finished painting, and one likely executed from observation. The beetle, structured, modular, and richly colored after the rotting matter it consumes, arches backward lifting the curve of its spiky mandibles. In this icon of natural design, the artist mimics nature not only with respectful attention to detail but also with talent at illusion: the shadow cast beneath the stationary armored trunk makes the beetle seem to strut across the canvas—just as the crablike claws make its harmless frame seem ferocious and menacing.

Dürer's realistic rendering of this humble bug is a tribute to the minutest in nature—that which is often overlooked or summarily destroyed, its importance lost to ignorance or neglect. Such is the case with the endangered stag beetle, thoroughly benign but seemingly ominous, all too readily squashed in its disappearing woodsy habitat.

Other critters, not so benign or visible, are also easy to ignore, their pestiferous history relegated to the past and quickly forgotten. Blood-thirsty ticks, bed bugs, and other insects, as if caught in some Gothic time machine, continue to torment humans, still claiming their lives, if not their souls. Renewed infestations of ticks causing meningoencephalitis in Germany (*9*) and of bed bugs compromising health in Canada and elsewhere (*10*) warn against ignorance and neglect regarding visible or invisible tiny creatures of nature.
